# New species of *Ehrlichia* isolated from *Rhipicephalus (Boophilus) microplus* shows an ortholog of the *E. canis* major immunogenic glycoprotein gp36 with a new sequence of tandem repeats

**DOI:** 10.1186/1756-3305-5-291

**Published:** 2012-12-11

**Authors:** Alejandro Cabezas Cruz, Erich Zweygarth, Mucio Flavio Barbosa Ribeiro, Julia Angelica Gonçalves da Silveira, Jose de la Fuente, Libor Grubhoffer, James J Valdés, Lygia Maria Friche Passos

**Affiliations:** 1 University of South Bohemia, Faculty of Science, České Budějovice, Czech Republic; 2 Comparative Tropical Medicine and Parasitology, Ludwig-Maximilians-Universität München, Munich, Germany; 3 Departamento de Parasitologia, ICB-UFMG, Belo Horizonte, Brazil; 4 Instituto de Investigación de Recursos Cinegéticos, IREC, Ciudad Real, Spain; 5 Department of Veterinary Pathobiology, Center for Veterinary Health Sciences, Oklahoma State University, Oklahoma, USA; 6 Departamento de Medicina Veterinaria Preventiva, INCT-Pecuária, Escola de Veterinária-UFMG, Belo Horizonte, Minas Gerais, Brazil

**Keywords:** *Ehrlichia* spp, *Rhipicephalus (Boophilus) microplus*, Phylogenetic analysis, Gp36 major immunogenic protein

## Abstract

**Background:**

*Ehrlichia* species are the etiological agents of emerging and life-threatening tick-borne human zoonoses that inflict serious and fatal infections in companion animals and livestock. The aim of this paper was to phylogeneticaly characterise a new species of *Ehrlichia* isolated from *Rhipicephalus (Boophilus) microplus* from Minas Gerais, Brazil.

**Methods:**

The agent was isolated from the hemolymph of *Rhipicephalus (B.) microplus* engorged females that had been collected from naturally infested cattle in a farm in the state of Minas Gerais, Brazil. This agent was then established and cultured in IDE8 tick cells. The molecular and phylogenetic analysis was based on *16S rRNA*, *groEL, dsb*, *gltA* and *gp36* genes. We used the maximum likelihood method to construct the phylogenetic trees.

**Results:**

The phylogenetic trees based on *16S rRNA*, *groEL, dsb* and *gltA* showed that the *Ehrlichia* spp isolated in this study falls in a clade separated from any previously reported *Ehrlichia* spp. The molecular analysis of the ortholog of *gp36,* the major immunoreactive glycoproteins in *E. canis* and ortholog of the *E. chaffeensis gp47,* showed a unique tandem repeat of 9 amino acids (VPAASGDAQ) when compared with those reported for *E. canis*, *E. chaffeensis* and the related mucin-like protein in *E. ruminantium*.

**Conclusions:**

Based on the molecular and phylogenetic analysis of the *16S rRNA*, *groEL, dsb* and *gltA* genes we concluded that this tick-derived microorganism isolated in Brazil is a new species, named *E. mineirensis* (UFMG-EV), with predicted novel antigenic properties in the *gp36* ortholog glycoprotein. Further studies on this new *Ehrlichia* spp should address questions about its transmissibility by ticks and its pathogenicity for mammalian hosts.

## Background

The emergence of multiple *Ehrlichia* species as etiological agents of newly discovered human zoonoses and the previous recognition of these agents as causing serious disease in companion animals and livestock have intensified the interest in these pathogens. Ehrlichiae are tick-transmitted obligate intracellular gram-negative bacteria that are maintained in nature by persistent infection of mammalian hosts [1]. They are microorganisms residing within the cytoplasmic vacuoles of monocytes, granulocytes, or platelets of humans and animals. *Ehrlichia* species elicit illnesses with fever, headache, leukopenia, and thrombocytopenia [2].

The obligately intracellular alpha-proteobacterial genus *Ehrlichia* (Rickettsiales: Anaplasmataceae) is spread all over the world and are comprised of five recognized species that are tick-transmitted, with three of the five causing human ehrlichiosis (*E. canis*, *E. chaffeensis*, and *E. ewingii*) [3]. The agent that causes the veterinary disease heartwater (*E. ruminantium*) can potentially infect humans [2,4] and *Ehrlichia muris* has not been associated with human infection. In addition, numerous candidate entities have been reported (“*E. walkerii*”, “*E. shimanensis*”, “*Ixodes ovatus ehrlichia*”, “*Panola Mountain ehrlichia*”, etc.), all isolated from hard ticks and mainly characterized by PCR sequencing [3]. To date, only three species of the genus *Ehrlichia* have been reported in Brazil: *E. canis*, *E. ewingii* and *E. chaffeensis*[5].

Different hard ticks species have been associated with transmitting members of the genus *Ehrlichia*: *Rhipicephalus sanguineus* and *Dermacentor variabilis* (*E. canis*), *Amblyomma americanum*[6] and *Dermacentor variabilis*[5] (*E. chaffeensis* and *E. ewingii*), *Haemaphysalis* spp and *Ixodes* spp (*E. muris*) and *Amblyomma* spp (*E. ruminantium*) [6].

Polyphasic taxonomy has been advocated to ensure well-balanced determination of taxonomic relationships [7]. Different genes have been proposed to classify ehrlichial agents. The most widely used are *16S rRNA*[8,9], *groESL* operon [10]*, groEL gene*[11]*, gltA*[7], *dsb*[12]*, gp36* and *gp19*[13]. The *gp36* belong to the group of major immunogenic antigen in *E. canis* (*gp36*) and *E. chaffeensis* (*gp47*) and both are orthologs to the mucin-like protein in *E. ruminantium*. These glycoproteins have tandem repeats that contain major B-cell epitopes with carbohydrate determinants, which contribute substantially to the immunoreactivity of these proteins. Only five types of tandem repeats have been characterized [14]. Of these glycoproteins, *gp36* is the most divergent gene among *E. canis* isolates [15]. Nevertheless, the tandem repeat is highly conserved among different isolates, changing only in the number of repeats [13] and in few amino acids among *E. canis* isolates [15].

Recently, we have isolated an organism from hemolymph of *R. (B.) microplus* engorged females which had been collected from naturally infested cattle in Brazil (unpublished data). This organism has been propagated continuously *in vitro*, both in a tick cell line (IDE8) and in a monocyte-macrophage cell line from a dog (DH82), and has been initially characterised as a new genotype of *Ehrlichia* spp (UFMG-EV strain) [16]. In the present study we report further molecular and phylogenetic analyses focusing on five genes (*16S rRNA*, *groESL*, *gltA*, *dsb* and *gp36*) of this new organism, from now on referred as *Ehrlichia mineirensis* (UFMG-EV).

## Methods

### Organism isolation and *in vitro* cultivation

Eleven *R. (B.) microplus* engorged females, larger than 4.5 mm in length, were collected from naturally infested calves (4 to 6 months old) from a farm in Minas Gerais, Brazil. The ticks were washed, blotted dry, and disinfected with Germekil (Johnson, Brazil.), for 30 minutes at room temperature. After several washes in sterile distilled water, the ticks were individually placed into polystyrene plates and were incubated at 27°C and relative humidity over 83%. After a 10-day incubation period hemolymph were collected to provide material for infecting IDE8 cells [17]. Each tick was held with sterile forceps, the cuticula was again sterilized, as previously described, and the leg cut with a sterile scalpel blade. The hemolymph was collected using a capillary tube to gather the draining fluid. Hemolymph from three ticks were pooled in a tube containing 200 μl of culture medium, which constitute the inoculum to infect one culture flask containing an on growing IDE8 cell monolayer.

After infection, the culture flask was monitored daily by examination of cytocentrifuge smears made from 50 μl aliquots taken from the culture suspension. Smears were fixed twice with methanol (for 10 min), stained with an 8% Giemsa solution for 30 min and examined under oil immersion at 1,000x magnification. The first infected cells were detected 28 days after culture initiation.

Maintenance of cultures was carried out with medium changes weekly. Briefly, IDE8 cells were maintained at 32°C in L-15B medium [18], supplemented with 5% heat-inactivated foetal bovine serum, 10% tryptose phosphate broth, 0.1% bovine lipoprotein concentrate (MP Biomedicals, Santa Ana, CA, USA), 100 IU/ml penicillin and 100 μg/ml streptomycin. Infected IDE8 cultures were propagated in a modified L-15B medium as outlined above, further supplemented with 0.1% NaHCO_3_ and 10 mM HEPES. The pH of the medium was adjusted to 7.5 with 1 N NaOH. Infected cultures were propagated at 34°C in 25 cm^2^ plastic culture flasks in 5 ml of the medium under normal atmospheric conditions.

### Genomic DNA isolation

The DNeasy Blood & Tissue Kit (Qiagen Inc. Valencia, Calif.) was used for extraction of DNA from infected IDE8 cells. DNA extraction was performed according to the manufacturer’s instructions. The extracted material was eluted from the columns in 100 μl of sterile double distilled H_2_O (ddH2O), and the DNA concentration and purity were determined by measuring the optical density at both 260 and 280 nm with a DNA-RNA calculator (NanoDrop® ND-1000, Peqlab, Erlangen, Germany). Ten-fold dilutions were done with the genomic DNA and separated in aliquot of 10 μl each and kept frozen until their use in a PCR reaction.

### PCR

The primers used in this study are shown in (Table 1). The oligonucleotide primers used for the amplification of *dsb* gene and *gltA* gene were designed for this study using primer design software (PrimerSelect; DNAStar, USA) and information from the *E. canis* genome [GenBank: CP000107] [19]. Two independent PCR reactions were performed for each gene. For each PCR amplification, 2 μL of extracted DNA was used as the template in a 25 μL reaction mixture containing 20pmol of each primer and 2X PCR Master Mix (Promega, USA). The reactions were conducted in an Eppendorf thermocycler (Eppendorf Mastercycler personal AG, 22331 Hamburg, Germany) according to the parameters: 2 min at 94°C followed by 40 cycles of 30 sec at 94°C, 1 min at 45°C, and 1.5 min at 72°C with a final extension step of 5 min. The PCR products were stained using an Ethidium bromide free system, 6X Orange DNA Loading Dye (Thermo Scientific, Germany) and visualized in 0.8% agarose minigels.

**Table 1:** **Primers used in this study for the amplification of the *16S rRNA, groESL, gltA, dsb *and *gp36 *genes from *E. mineirensis *(UFMG-EV) genomic DNA**

**Target**	Primers*	**Sequence**	**Expected size (Kb)**
***16Sr RNA***	8 F^9^	5^′^- AGTTTGATCATGGCTCAG – 3^′^	1.4
	1448R	5^′^- CCATGGCGTGACGGGCAGTGTG – 3^′^	
***groEL***	HS1^10^	5^′^- TGGGCTGGTA(A/C)TGAAAT – 3^′^	1.4
	HS6	5^′^- CCICCIGGIACIA(C/T)ACCTTC – 3^′^	
***gltA***	gltAF1	5^′^- CTTCTGATAAGATTTGAAGTGTTTG – 3^′^	1.5
	gltAR1	5^′^- CTTTACAGTACCTATGCATATCAATCC – 3^′^	
***dsb***	dsbF2	5^′^- CTTAGTAATACTAGTGGCAAGTTTTCCAC – 3^′^	0.683
	dsbR2	5^′^- GTTGATATATCAGCTGCACCACCG – 3^′^	
***gp36***	EC36-F1^13^	5^′^- GTATGTTTCTTTTATATCATGGC – 3^′^	1.0
	EC36-R1	5^′^- GGTTATATTTCAGTTATCAGAAG – 3^′^	

*Primers F are forward and R reverse.

### Cloning and sequencing

The resulting PCR products were electrophoresed on a 0.8% agarose gel. The size of the amplified fragments was checked by comparison to a DNA molecular weight marker (100-bp DNA Ladder; Promega, USA). In each case, the single amplified product of the expected size was column purified using the QIAquick PCR Purification Kit (Qiagen, USA) and then ligated into the TOPO TA Cloning Kit (Invitrogen, USA) for subsequent transformation in *Escherichia coli* TOP 10 Chemically Competent cells. For each gene, five individuals clones containing the cloned fragment in the TOPO vector were purified using the QIAprep Spin Miniprep Kit (Qiagen, USA) and prepared for sequencing using an ABI 3130 sequencer (Applied Biosystems, USA) and the Big Dye Terminator v3.1 Cycle Sequencing Kit (Applied Biosystems, USA) with the M13F and M13R vector primer. Both the sense and antisense strands of each PCR-amplified product were sequenced, and the sequences were then manually edited to resolve any ambiguities. A consensus sequence was obtained for each amplified PCR product by comparing both the sense and antisense sequences from the five clones.

### DNA sequence analysis

To find the homology of our sequences we used the database Nucleotide collection (nr/nt) using Megablast (optimize for highly similar sequences) from the BLAST server [20]. Nucleotide sequences were aligned using BLAST [20] and protein sequences were aligned using the multiple-alignment program CLUSTALW [21]. The homology between sequences was analyzed using MegAlign, DNAStar, USA. Nucleotide sequences were translated to amino acid (aa) sequence by the ExPASy translation tool of the Swiss Institute of Bioinformatics [22].

The phylogenetic analysis was performed as follows: sequences were aligned with MUSCLE (v3.7) configured for highest accuracy [23]. After alignment, ambiguous regions (i.e., containing gaps and/or poorly aligned) were removed with Gblocks (v0.91b) [24]. The phylogenetic tree was reconstructed using the maximum likelihood method implemented in the PhyML program (v3.0 aLRT) [25,26]. Reliability for internal branch was assessed using the bootstrapping method (100 bootstrap replicates). Graphical representation and edition of the phylogenetic tree were performed with TreeDyn (v198.3) [27]. The nomenclature used in the trees is according to Dumler *et al.*, [19]. The same analysis of similarity and phylogenetic relationships was performed for the genes *16S rRNA*, *groEL*, *gltA* and *dsb* with the exception that the *dsb* tree is unrooted and the rest are rooted.

### Analysis of the glycoprotein gp36 gene and putative aa sequence

The *gp36* ortholog was tested for the presence of signal peptide sequences with the computational algorithm SignalP trained on gram-negative bacteria [28]. The *gp36* protein sequence was evaluated for potential mucin-type O-linked glycosylation on serines and threonines with the computational algorithm NetOGlyc v3.1 [29] and for N-linked glycosylation was used the NetNGlyc 1.0 Server [30]. The Tandem Repeats Finder database [31] was used to analyze the tandem repeats. The prediction of continuous B cell epitopes was done using the B cells Epitopes Prediction Tool [32] and the 3D structure of the glycoprotein and the predicted epitopes was obtained using the algorithm contained in the ElliPro epitope modeling tool and sequences available in the ElliPro server [33]. As previously reported [14], for the convenience of sequence comparison the *gp36* gene orthologs were divided into three regions: 5’ end pre-repeat region, a tandem repeat region, and 3’ end post-repeat region.

### Sequences used in this study

The sequences obtained from *Ehrlichia mineirensis* (UFMG-EV) have been deposited in GeneBank, and their accession numbers are: *16S rRNA* [GenBank: JX629805], *groESL* [GenBank: JX629806], *dsb* [GenBank: JX629808], *gltA* [GenBank: JX629807] and *gp36* [GenBank: JX629809]. The *16S rRNA*, *groEL*, *gltA, dsb* and *gp36* sequences used for the phylogenetic tree or molecular analysis in general were obtained from GenBank and their accession numbers are show in the Tables and Figures where they have been mentioned.

## Results

### Sequence analysis of *16S rRNA*

**Figure 1:**
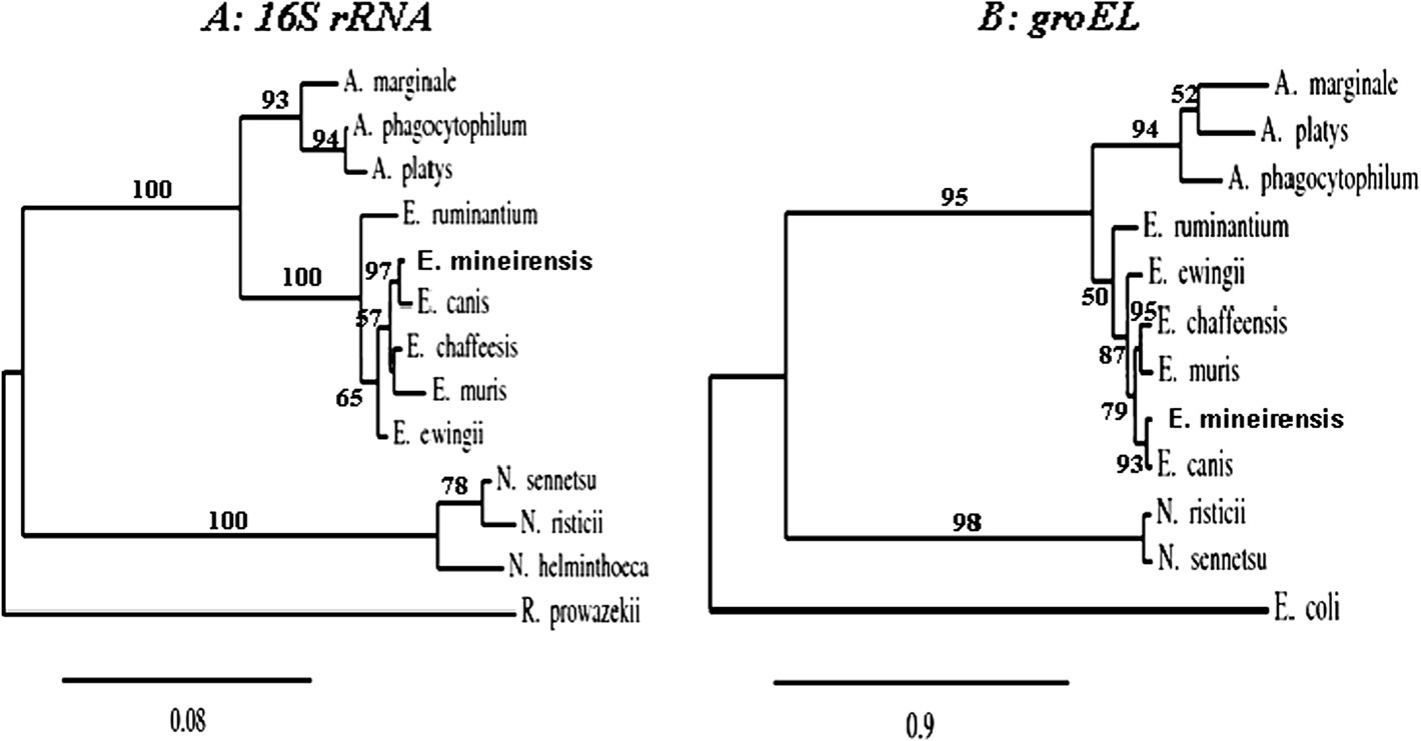
**AB Phylogenetic trees based on the *16S rRNA *(A) and *groEL *(B) genes sequences from members of the family Anaplasmataceae.** The tree shows that *E. mineirensis* (UFMG-EV) falls in a clade separated from all the previous reported sequences. Bootstrap values are shown as % in the internal branch. Only bootstrap values equal or higher than 50% are shown. *Rickettsia prowazekii 16S rRNA* sequence was used to root the *16S rRNA* tree and *E.coli groEL* gene was used to root the *groEL* tree. The GenBank accession numbers of the sequences used to build the *16S rRNA* tree are: *E. muris*, AB013008; *E. chaffeensis*, AF147752; *E. ruminantium*, AF069758; *E. ewingii*, U96436; *A. marginale*, M60313; *A. phagocytophilum*, M73224; *A. platys*, M82801; *N. helminthoeca*, U12457; *N. sennetsu*, M73225; *N. risticii*, AF036649; *E. canis*, GU810149; *R. prowazekii*, NR044656. The GenBank accession numbers of the sequences used to build the *groEL* tree are: *E. muris*, AF210459; *E. chaffeensis*, L10917; *E. ruminantium*, U13638 ; *E. ewingii*, AF195273; *A. marginale*, AF165812; *A. phagocytophilum*, U96729; *A. platys*, AY008300; *N. sennetsu*, U88092; *N. risticii*, U96732; *E. canis*, U96731; *E. coli*, X07850.

In order to obtain relevant information from *16S rRNA* at the species level, the primers 8 F and 1448R were used to isolate a fragment of ~1.4Kb. Approximately a 1.4Kb amplicon corresponding to the expected size of targeted *16S rRNA* gene fragment was obtained (data not shown). A consensus sequence of 1.384 Kb was obtained from 2 independent PCRs and five clones were sequenced. In total, our sequence had 10 changes of nucleotides when compared with *E. canis* [GenBank: GU810149] with two insertions and three deletions (data not shown). The percent of identities with all the members of the *Ehrlichia* genus are shown in the Table 2 upper triangle. Figure 1A shows the tree build using the maximum likelihood method; it shows that *E. mineirensis* (UFMG-EV) falls in a clade separated from all the previous reported sequences. The tree build with the neighbour joining method using the Kimura 2 parameters substitution model show identical results (data not shown).

**Table 2:** **Identities comparison of *16S rRNA *and *dsb *genes between *E. mineirensis *(UFMG-EV) and other members of the genus *Ehrlichia***

Percent of nucleotide similarity of 16S rRNA*						
	***Ehrlichia mineirensis *(UFMG-EV)**	***E. canis *[GU810149]**	***E. chaffeensis *[AF147752]**	***E. ewingii *[U96436]**	***E. muris *[AB013008]**	***E. ruminantium *[AF069758]**
***Ehrlichia mineirensis *(UFMG-EV)**	***	98.3 (*16SrRNA)*	96.9 (*16SrRNA)*	96.4 (*16SrRNA)*	94.5 (*16SrRNA)*	95.0 (*16SrRNA)*
***Ehrlichia canis *[AF403710]**	94.7 (*dsb*)	***	98.4 (*16SrRNA)*	97.9 (*16SrRNA)*	97.1 (*16SrRNA)*	97.2 (*16SrRNA)*
***Ehrlichia chaffeensis *[AF403711]**	82.3 (*dsb*)	83.5 (*dsb*)	***	98.1 (*16SrRNA)*	97.6 (*16SrRNA)*	96.9 (*16SrRNA)*
***Ehrlichia ewingii *[AY428950]**	78.6 (*dsb*)	76.9 (*dsb*)	78.0 (*dsb*)	***	97.2 (*16SrRNA)*	97.1 (*16SrRNA)*
***Ehrlichia muris *[AY236484]**	81.1 (*dsb*)	81.1 (*dsb*)	84.5 (*dsb*)	77.2 (*dsb*)	***	96.4 (*16SrRNA)*
***Ehrlichia ruminantium *[AF308669]**	76.9 (*dsb*)	74.6 (*dsb*)	77.1 (*dsb*)	76.6 (*dsb*)	76.4 (*dsb*)	***

Percent of nucleotide similarity of dsb*.

*The values are % of nucleotide sequence similarity for 1.3Kb (16Sr RNA) and determined from pairwise aligment using DNASTAR software (MegAlign; DNASTAR, Inc., Madison, WI).

Accession Numbers are from GenBank.

**Figure 2:**
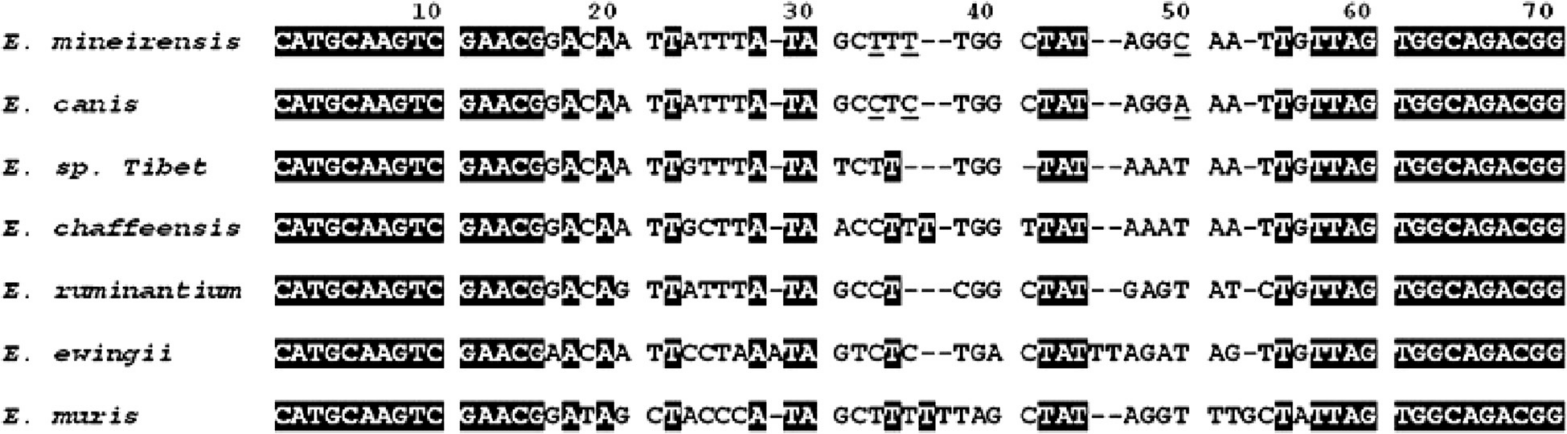
**A highly variable region of sequence located at 5’ end of the *16S rRNA *gene revealed by multiple alignments of *16S rRNA *gene sequences of *Ehrlichia *genus.** Underlined are the nucleotide differences found between *E. canis* and *E. mineirensis* (UFMG-EV). The GenBank accession numbers of the sequences show in the alignment are: *E. muris,* AB013008*; E. chaffeensis,* AF147752*; E. ruminantium*, AF069758*; E. ewingii*, U96436 and *E. canis,* GU810149.

The gene *16S rRNA* has a highly variable region located at the 5’ end of the gene [8]. This fragment is useful in identifying *Ehrlichia* spp [9]. Figure 2 shows three changes in nucleotides in *E. mineirensis* (UFMG-EV) in comparison with *E. canis* and seven changes in nucleotides when compare with *Ehrlichia. sp. Tibet* which was isolated from *R. microplus*[8].

### Sequence analysis of *dsb*

**Figure 3:**
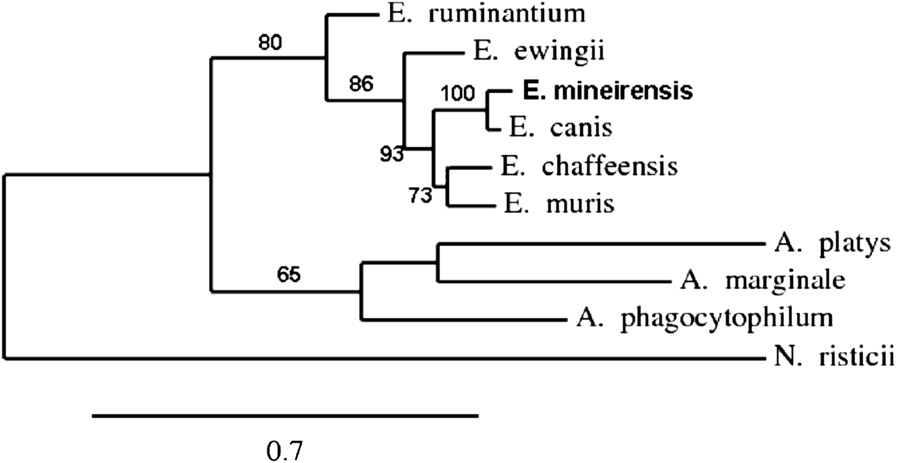
**Phylogenetic unrooted tree based on the *dsb *gene sequences from members of the family Anaplasmataceae.** The tree shows that *E. mineirensis* (UFMG-EV) falls in a clade separated from all the previous reported sequences and the previously reported *E. canis* dsb sequences. Bootstrap values are show as% in the internal branch. Only bootstrap values equal or higher than 50% are shown. The GenBank accession numbers of the *dsb* sequences used to build the tree are: *E. canis*, AF403710; *E. canis Uberlandia*, GU586135; *E. canis Jaboticabal*, DQ460716; *E. canis Sao Paulo*, DQ460715; *E. muris*, AY236484; *E. chaffeensis*, AF403711; *E. ruminantium*, AF308669, clon 18hw; *E. ewingii*, AY428950.

The amplicon obtained from the PCR set up with the primers dsbF2 and dsbR2 gave a band with the expected size of 0.7 Kb. A fragment of 0.683 Kb of the gene *dsb* was obtained and sequenced. *Dsb* gene sequences for available *Ehrlichia* spp. were aligned using clustalW. The alignment shows that *dsb* gene is conserved (76.4% - 94.7%) within the genus (Table 2 lower triangle). The aa sequence shows homology from 72.0% to 95.0% with *E. ruminantium* [GenBank: AF308669, clon 18hw] and *E. canis* [GenBank: AF403710], respectively. When compared with the complete dsb from *E. canis* [AF403710] 10 aa changes are observed (data not shown). The changes are concentrated at the carboxyl-terminus of the protein. Different dsb isolates of *E. canis* share 100% of identity among them (Table 3) The phylogenetic tree shows that *E. mineirensis* (UFMG-EV) *dsb* is separated from its homologs in other species of the *Ehrlichia* genus (Figure 3).

**Table 3:** **Unique aa changes in the carboxyl terminal of *Ehrlichia mineirensis *(UFMG-EV) dsb differ from *E. canis *dsb available in the GenBank**

**Isolates**	**aa position**^**1**^						
	**Identity %**^**1**^	**160**	**162**	**168**	**184**	**185**	**204**
***Ehrlichia canis *[AF403710]**	100	V	Q	H	H	Y	**T**
***Ehrlichia canis *Uberlandia [GU586135]**	100	.	.	.	.	.	**.**
***Ehrlichia canis *Sao Paulo [DQ460715]**	100	.	.	.	.	.	**.**
***Ehrlichia canis *Jaboticabal [DQ460716]**	100	.	.	.	.	.	**.**
***Ehrlichia mineirensis *(UFMG-EV)**	94.0	A	K	Y	N	H	**A**

1- Positions and % of identities are based on the sequence of *E. canis* [GenBank: AF403710]. The dots below the aa letters mean conserved positions.

Accession Numbers are from GenBank.

### Sequence analysis of *groESL* operon

The amplification with primers HS1-HS6 produced a PCR product in the expected size 1.4Kb. The nucleotide sequences of the PCR products amplified from *E. mineirensis* (UFMG-EV) contained a reading frame corresponding to the 26 aa carboxyl-terminus of *groES*, 416 aa of the amino-terminal end of *groEL*, and the spacer between them. The length of the nucleotide sequence of the spacer region in the sequence reported here were 95 bases. Sequence homology analyses were done for each of the nucleotide sequences and the deduced aa sequences from the partial GroES and GroEL reading frames. Nucleotide and aa sequence homologies with other members of the *Ehrlichia* genus are presented in Table 4. A phylogenetic tree based on multiple sequence alignment of the 1.249 Kb corresponding to *groEL* is presented in Figure 1B.

**Table 4:** **Identities comparison of *groEL *gene and putative aa sequence between *Ehrlichia mineirensis *(UFMG-EV) and other members of *Ehrlichia *genus**

Percent of nucleotide (nt) similarity*						
	***E. mineirensis *(UFMG-EV)**	***E. canis***	***E. chaffeensis***	***E. ewingii***	***E. muris***	***E. ruminantium***
***Ehrlichia mineirensis *(UFMG-EV)**	***	97.2 (nt)	92.3 (nt)	91.0 (nt)	92.0 (nt)	87.3 (nt)
***Ehrlichia canis [U96731]***	99.0 (aa)	***	92.5 (nt)	90.9 (nt)	92.4 (nt)	87.6 (nt)
***Ehrlichia chaffeensis [L10917]***	97.0 (aa)	97.0 (aa)	***	91.7 (nt)	94.3 (nt)	87.8 (nt)
***Ehrlichia ewingii [AF195273]***	95.0 (aa)	95.0 (aa)	96.0 (aa)	***	91.5 (nt)	88.0 (nt)
***Ehrlichia muris [AF210459]***	97.0 (aa)	97.0 (aa)	99.0 (aa)	97.0 (aa)	***	87.3 (nt)
***Ehrlichia ruminantium [U13638]***	92.0 (aa)	92.0 (aa)	93.0 (aa)	92.0 (aa)	93.0 (aa)	***

Percent of amino acid (aa) similarity*.

*The values showed are % of nucleotide and aa sequence similarity of 1.249 Kb determined from pairwise aligment using DNASTAR software (MegAlign; DNASTAR, Inc., Madison, WI) and 416 aa of the amino terminal determined from ClustalW.

Accession Numbers are from GenBank.

### Sequence analysis of *gltA gene*

**Figure 4:**
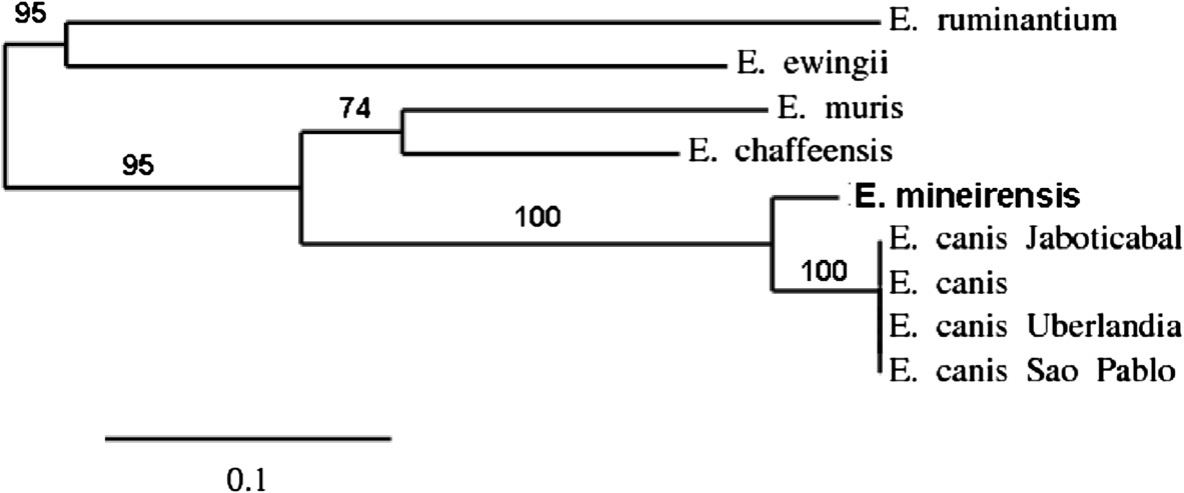
**Phylogenetic tree based on the citrate synthase *(gltA) *gene sequences from members of the family Anaplasmataceae.** The tree shows that *E. mineirensis* (UFMG-EV) falls in a clade separated from all the previously reported sequences. Bootstrap values are show as % in the internal branch. Only are showed bootstrap values equal or higher than 50%. *N. risticii gltA* sequence was used to root the tree. The GenBank accession numbers of the *gltA* sequences used to build the tree are as follow: *E. canis*, AF304143; *E. muris*, AF304144; *E. chaffeensis*, AF304142; *E. ruminantium*, AF304146; *E. ewingii*, DQ365879; *A. marginale*, AF304140; *A. phagocytophilum*, AF304138; *A. platys*, AY077620.

Primers gltAF1 and gltAR1 were designed in this study using information from *E. canis* genome [GenBank: CP000107] and *E. chaffeensis gltA* gene sequence [GenBank: AF304142]. The full length of *gltA* gene of *E. mineirensis* (UFMG-EV) was isolated. A single band of ~1.5Kb was obtained from the PCR reaction (data not shown). The full length gene of 1.251 Kb was obtained after sequencing and consensus analysis. The putative citrate synthase protein predicted using the *E. mineirensis* (UFMG-EV) *gltA* gene was 416 aa. Table 5 shows the nucleotide and the aa similarities with other members of the *Ehrlichia* genus. The *gltA* gene has been proposed as an alternative tool for the phylogenetic analysis of the genus *Ehrlichia*[7]. Using the maximum likelihood method we built a phylogenetic tree showing that *E. mineirensis* (UFMG-EV) falls in a clade apart from any previously reported *gltA* genes in the family Anaplasmataceae (Figure 4).

**Table 5:** **Identities comparison of *gltA *gene and putative aa sequence between *E. mineirensis *(UFMG-EV) and other members of *Ehrlichia *genus**

Percent of nucleotide (nt) similarity*						
	***E. mineirensis *(UFMG-EV)**	***E. canis***	***E. chaffeensis***	***E. ewingii***	***E. muris***	***E. ruminantium***
***Ehrlichia mineirensis *(UFMG-EV)**	***	94.3 (nt)	84.6 (nt)	80.9 (nt)	84.8 (nt)	77.6 (nt)
***Ehrlichia canis *[AF304143]**	94.0 (aa)	***	85.0 (nt)	82.2 (nt)	85.4 (nt)	79.0 (nt)
***Ehrlichia chaffeensis *[AF304142]**	82.0 (aa)	84.0 (aa)	***	82.0 (nt)	87.0 (nt)	78.9 (nt)
***Ehrlichia ewingii *[DQ365879]**	79.0 (aa)	80.0 (aa)	77.0 (aa)	***	82.5 (nt)	79.4 (nt)
***Ehrlichia muris *[AF304144]**	82.0 (aa)	84.0 (aa)	85.0 (aa)	78.0 (aa)	***	79.6 (nt)
***Ehrlichia ruminantium *[AF304146]**	74.0 (aa)	77.0 (aa)	75.0 (aa)	75.0 (aa)	77.0 (aa)	***

Percent of aa similarity*.

*The values showed are % of nucleotide and aa sequence similarity of the full length determined from pairwise aligment using DNASTAR software (MegAlign; DNASTAR, Inc., Madison, WI) and the putative encoded aa determinated from ClustalW.

Accession Numbers are from GenBank.

### Sequence analysis of the *gp36* gene and the putative encoded protein sequence

The *gp36* based PCR products derived from the isolate reported here had a molecular size of 1000 base pair (bp) (data not shown). Subsequent cloning of the PCR amplicons followed by sequencing showed that our gene was 0.948 Kb encoding a predicted protein with 315 aa and a molecular mass of 31.51 KDa (28.89 KDa without the predicted 23-aa signal peptide). We found that the gp36 protein isolated in our study is a putative glycoprotein. The aa sequence of gp36 in our study has five potential sites of O-glycosylation and two of N-glycosylation. The O-carbohydrates were predicted to be linked to three serines (S) of the tandem repeat region at position 155, 164 and 173 and two threonines (T) present in the post-repeat region at position 286 and 289. We explored as well the possibility to find N-glycosylation on putative glycosylated asparagines (N). Two sequons of N-glycosylation (N-Xaa-T/S) at the pre-repeat region were found: NRS (at position 81) and NFS (at position 106).

### Differences found in the Region I (The 5′ end pre-repeat region)

Alignment of the *gp36* ortholog obtained in this study revealed that our sequence was 422 nucleotides in length encoding for 141 aa (Table 6). The nucleotide and predicted aa sequences exhibited relatively low identities, ranging from 54.9% to 91.2%, and from 38.0% to 82.0%, respectively, in comparison with related genes previously published for the *gp36* orthologs in *E. canis*, *E. chaffeensis* and *E. ruminantium*[14] (Table 6).

**Table 6:** **Length and percent of nucleotide and aa homology of the 5’ end pre-repeat region between the orthologs of *gp36 *in *Ehrlichia mineirensis *(UFMG-EV) and related genes**

		**Nucleotide**		**aa**	
**Source**	**Strain**	**Length**^**1**^	**Homology**^**2**^	**Length**^**3**^	**Homology**^**4**^
***Ehrlichia mineirensis***	(UFMG-EV)	422	-	141	-
***Ehrlichia canis gp36***	TWN1 [EF551366]	425	91.2	142	82
	Louisiana [DQ146151]	428	88.2	143	78
	Sao Paulo [DQ146154]	428	88.4	143	78
	Cameroon [DQ146155]	428	88.6	143	79
***Ehrlichia chaffeensis gp47***	Arkansas [DQ085430]	471	61.8	157	52
	Sapulpa [DQ085431]	461	62.1	154	53
	Jax [DQ146156]	461	60.7	154	51
	St Vincent [DQ146157]	461	62.1	154	53
***Ehrlichia ruminantium *mucin-like protein**	Highway [AF308673]	410	54.9	137	38

1 - The length were determinate using the Tandem Repeats Finder database [30].

2 - Percent of nucleotide homology were calculated with MegAlign, DNAStar, USA. Comparing with *E. mineirensis* (UFMG-EV).

3 - The length was determined using ClustalW [20] in comparison with *Ehrlichia mineirensis* (UFMG-EV).

4 - Percent of aa homology were calculated with ClustalW [20]. Comparing with *E. mineirensis* (UFMG-EV).

Accession Numbers are from GenBank.

### Region II (the tandem repeat region)

Region II in *E. mineirensis* (UFMG-EV) contains 16 tandem repeats of 27 bp, each encoding nine aa. The single tandem repeat had the sequence VPAASGDAQ and was completely different to the sequences reported for glycoprotein orthologs of *gp36 E. canis*, *gp47 E. chaffeensis* and *E. ruminantium* mucin-like protein (Table 7). The tandem repeat of *E. mineirensis* (UFMG-EV) is a serine enriched area of the total protein sequence but does not contain threonine. Its glycoprotein gene shows a high C + G percent in the whole gene (42.0%) and in the tandem repeat region (52.1%).

**Table 7:** **Summary of*Ehrlichia*tandem repeats present in gp36 glycoprotein orthologs**

		**Repeat**			
**Source**	**Strain**	**Length(bp)**^**1**^	**No.**^**1**^	**Homology% (bp)**^**1**^	**Consensus tandem repeat sequence(aa)**^**2**^
***Ehrlichia mineirensis***	(UFMG-EV)	27	16.0	100	VPAASGDAQ
***Ehrlichia canis gp36***	TWN1 [EF551366]	27	13.2	100	TEDSVSAPA
	Louisiana [DQ146151]	27	5.2	99	. . . . . . .
	Sao Paulo [DQ146154]	27	18.2	100	. . . . . . .
	Cameroon [DQ146155]	27	16.2	100	. . . . . . .
	IS [EF636663]	27	11.2	99	TEDPVSATA
***Ehrlichia chaffeensis gp47***	Arkansas [DQ085430]	57	7.0	99	ASVSEGDAVVNAVSQETPA
	Sapulpa [DQ085431]	99	4.5	99	EGNASEPVVSQEAAPVSESGDAANPVSSSENAS
	Jax [DQ146156]	99	4.5	98	. . . . . . . . . . . . . . . . . . . . . . . .
	St Vincent [DQ146157]	99	3.4	98	. . . . . . . . . . . . . . . . . . . . . . . .
***Ehrlichia ruminantium*mucin-like protein**	Highway [AF308673]	27	21.7	99	VTSSPEGSV
	Welgevonden [CR767821]	27	56.0	95	. . . . . . .
	Gardel [CR925677]	66	16.9	99	SSEVTESNQGSSASVVGDAGVQ

1 – The length (bp), No of nucleotide repeats and the % of Homology were determinate using the Tandem Repeats Finder database [21].

2 – The dots below the tandems mean conserved aa sequence.

Accession Numbers are from GenBank.

### Region III (the 3′ end post-repeat region)

The comparison of region III among the orthologs show that it is a quite variable region, presenting differences in length, nucleotide and aa sequence. It has been widely revised by [14] and [15]. Our sequence was 94-bp length, which differ from any previously reported (data not shown). The percent identities of nucleotide and aa sequence in this region when compare with *E. mineirensis* (UFMG-EV) go from 12.2% (*E. chaffeensis* St Vincent*,* DQ146157) to 75% *(E. canis* TWN1*,* EF551366*)* and from 10% (*E. chaffeensis* St Vincent) to 32% (*E. canis* TWN1), respectively. *E. ruminantium* Highway mucin-like protein has 37.3% (bp) and 21% (aa) of homology with *E. mineirensis* (UFMG-EV).

### B cell epitopes analysis

**Figure 5:**
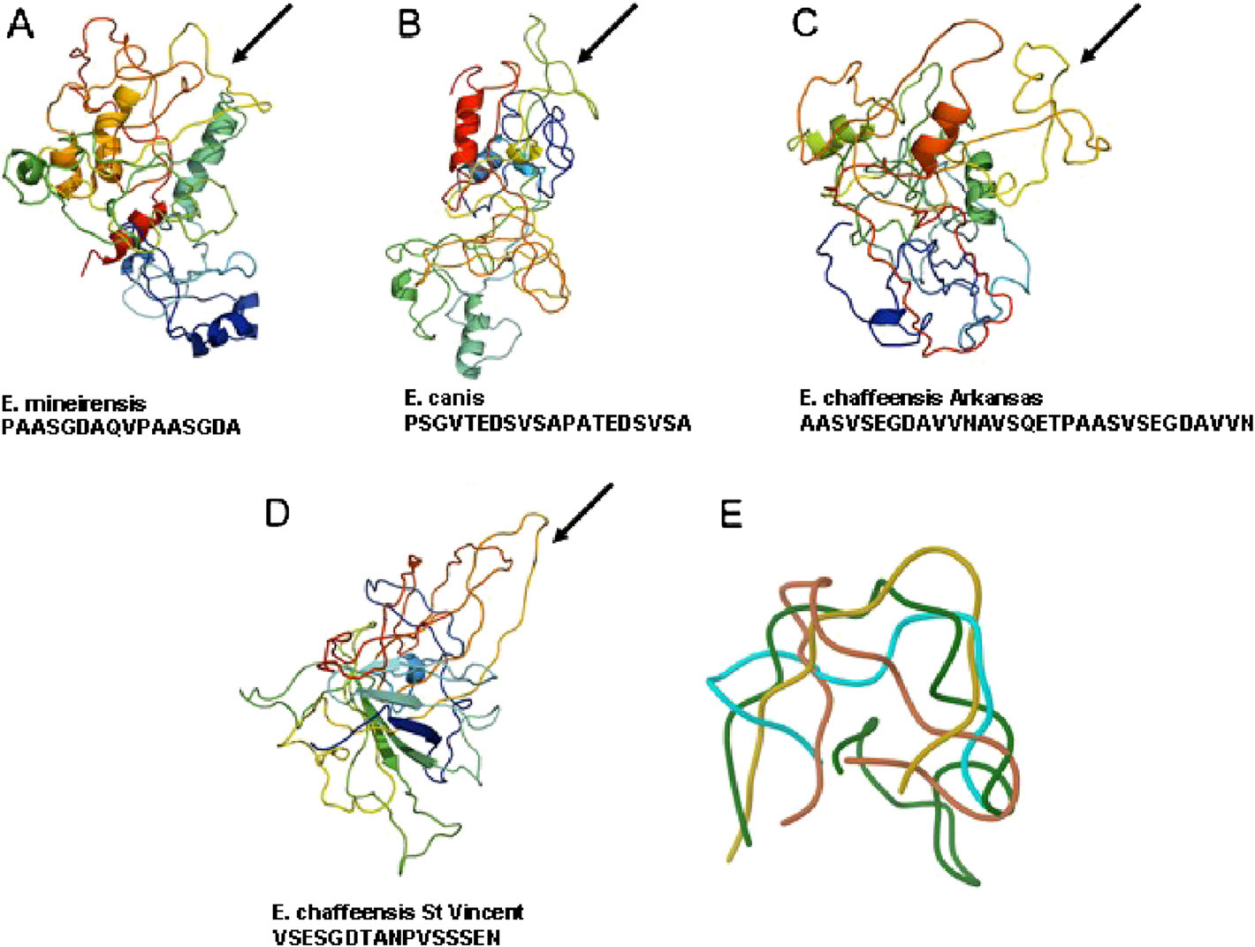
**A-E Epitope identification.** The modeled 3D structures for *E. mineirensis* (UFMG-EV) (**A**), *E. canis* (**B**; GenBank: EF560599), and *E. chaffeensis* (**C** and **D**; GenBank: DQ085430, DQ146157, respectively) depict the position of the predicted epitope (→). Protein structures are colored from blue (N-terminus) to red (C-terminus) according to the residue position. An epitope Cα superimposition (**E**) of *E. mineirensis* (UFMG-EV) (cyan), *E. canis* (brown), *E. chaffeensis* (GenBank: DQ085430; green) and *E. chaffeensis* (GenBank: DQ146157; yellow) depicting the differences in their overall structures, *E. mineirensis* (UFMG) having a 5-6 Å difference compared with the other epitopes).

The presence of B cell epitopes in the putative gp36 protein was predicted. The presence of one continuous B cell epitope was predicted in a highly hydrophobic repeat tandem region of our protein (197–212). Considering that gp36 (*E. canis*) and gp47 (*E. chaffeensis*) were the closest orthologs, we attempted to find B cell epitope in the tandem repeat of these species using the same algorithm employed for *E. mineirensis* (UFMG-EV). We found the presence of continuous B cell epitopes in the tandem repeat of *E. canis* gp36 [GenBank: EF560599] and *E. chaffeensis* gp47 [strain Arkansas, DQ085430 and strain St. Vincent, DQ146157]. The continuous epitopes found in these last three sequences were localized between the aa position 139–158, 195–225 and 203–218, respectively. The corresponding primary structures of the epitopes are shown in Figure 5A-E. We then compared the predicted 3D structures of the epitopes found in the gp36 orthologs in *E. mineirensis* (UFMG-EV), *E. canis* and the two from different strains of *E. chaffeensis*. We found that all epitopes were exposed on the surface of the predicted 3D structure of each protein. The superposition analysis of the epitopes 3D structure showed that they were structurally dissimilar with a root mean square deviation (rmsd) of 5-6 Å between the epitope of *E. mineirensis* (UFMG-EV) and others three Figure 5A-E. A linear correlation between the rmsd and % (dis)similarities among structure and sequences, respectively, is a valid interpretation for the evolution of homolog proteins [34]. Correlation for the epitopes of *E. mineirensis* (UFMG-EV) when compared with the other three orthologs gives an R^2^ = 0.77.

## Discussion

Polyphasic taxonomy has been advocated to ensure well-balanced determinations of taxonomic relationships [7]. Different genes have been proposed to classify ehrlichial agents, however, the most widely used are *16S rRNA*[8,9], *groESL* operon [10]*, groEL* gene [11]*, gltA*[7], *dsb*[12]*, gp36,* and *gp19*[13].

Sequence comparison of the *16S rRNA* gene is recognized as one of the most powerful and precise methods for determining the phylogenetic relationships of bacteria [8,11,35]. Our results were consistent with previous phylogenetic analysis of *Ehrlichia* spp by using the *16S rRNA* gene sequences [9,36]. In this study, our analysis of a relevant fragment of *16S rRNA* sequences revealed that the novel agent found in Brazilian *R. (B.) microplus* ticks was closely related to *E. canis* [GenBank: GU810149], but was also closely related to *E. chaffeensis* [GenBank: AF147752] showing 98.3% and 96.9% of homology, respectively. It is worth noting that the hypervariable region *16S rRNA* is well conserved in members of the same species (data not shown) and are different among members of *Ehrlichia* genus [8,9]. However, our hypervariable region of *16S rRNA* was different when compared with other members of *Ehrlichia* genus.

Since the *16S rRNA* gene is known to exhibit a high level of structural conservation with a low evolutionary rate, levels of sequence divergence greater than 0.5% in comparisons with nearly complete *16S rRNA* gene sequences of members of the genus *Ehrlichia* have been considered sufficient to classify organisms as different species [8,35]. The levels of divergence of the *16S rRNA* sequence between this novel Brazilian ehrlichial agent and the closest member of the Anaplasmataceae, *E. canis* was 1.7% in pairwise comparisons of 1384 base sequences (data not shown), and this level of difference should be sufficient to classify the novel ehrlichial agent as a new species of the genus *Ehrlichia*. Furthermore, the *16S rRNA* phylogenetic tree constructed with a maximum likelihood method show that *E. mineirensis* (UFMG-EV) falls in a different clade separated from any previously reported *Ehrlichia* spp.

The genes *groEL*[11] and *gltA*[7] have been proposed as an alternative to *16S rRNA* for the phylogenetic analysis of the Anaplasmatacaea family as they are less conserved than *16S rRNA* among the family members [7] and *dsb* gene has been previously used to classified members of the *Ehrlichia* genus [12]. It is important to note that the spacer of the *groESL* operon was 95 bp in *E. mineirensis* (UFMG-EV), which differs from the reported for *E. canis*, *E. chaffeensis*, *E. ruminantium* with 93, 100 and 96 bp, respectively [10]. The *gp36* orthologs are a divergent gene in *E. canis*, *E. chaffeensis* and *E. ruminantium* due to their high evolutionary pressure [14,15]. This gene has been used to differentiate new isolates of *E. canis* where *16S rRNA* was not well suited to discriminate between *E. canis* isolates [13].

In our study the level of similarity among ehrlichial *gltA* and *dsb* were lower than that of *16S rRNA* and *groEL* gene sequences in the genus *Ehrlichia*. *E. canis* was the closest *Ehrlichia* species to *E. mineirensis* (UFMG-EV) in all the studied genes. Similar phylogenetic relationships are observed between other members of the *Ehrlichia* genus – i.e., *E. chaffeensis/E. muris*, *N. risticii/N. sennetsu* and *A. marginale/A. platys*.

The architecture of *gltA*, *groEL* and *dsb* based phylogenetic trees were similar to that of the tree derived from the *16S rRNA* gene sequences. However, the trees constructed from *gltA* and *dsb* show more divergence than that from the *16S rRNA* and *groEL* gene. The difference of *E. canis* and *E. mineirensis* (UFMG-EV) was well established in all the four trees based on nucleotide sequences. *E. mineirensis* (UFMG-EV) was well defined, with higher bootstrap values in the *gltA* (100) and *dsb* (100) based trees than for those of the *16S rRNA* (97) and *groEL* (93) based tree.

Based on aa homology and genomic synteny analyses, it has been determined that the mucin-like protein of *Ehrlichia ruminantium*, *gp36* of *E. canis* and *gp47* of *E. chaffeensis* are orthologs [14]. Identity of 87.2% has been found in the pre-repeat region among geographically distant *E. canis* isolates [13]. The single tandem repeat was highly conserved among isolates (TEDSVSAPA) with variations in the number of repeats [13-15] and few conservative changes in amino acid sequences [15]. The tandem repeat genetic unit varies in length (from 27 bp – 99 bp) among the different orthologs, number of repeats (from 3.4 - 56) and the homology of the nucleotide and the aa sequence encoded in the repeat (Table 7). Our sequence contains a tandem repeat that shares an extremely low homology with the gp36 orthologs reported until now ranging from 22% (*E. ruminantium* and *E. canis)* to 33% (*E. chaffeensis*). Doyle *et al.*[14] describes *gp36* and *gp47* as glycoprotein sharing O-glycosylation predicted sites in the serines and threonines of the tandem repeat. It is noteworthy that the tandem repeat of our sequence does not contain threonine; nevertheless, we predicted three sites of O-glycosylation in the serines of the tandem repeat and two in threonines of the post-repeat region. Two N-glycosylation sites were found in our aa sequence. The analysis for N-glycosylation was done for *E. ruminantium*, *E. canis* and for *E. chaffeensis* ortholog sequences (data not shown) and potential sites of N-glycosylation were found as well for these sequences. Glycosylation plays a crucial role in the immunogenicity of these glycoproteins [14,15]. Deglycosylation of the *gp36* tandem repeat drastically reduces its immunogenicity [14]. Both *gp36* and *gp47* are described as the major immunoreactive protein of *E. canis* and *E. chaffeensis* and the tandem repeats contain the major antibody epitope [14,15]. It was found that the tandem repeat of gp36 from *E. mineirensis* (UFMG-EV) contain the major B cell epitope previously reported for the glycoprotein orthologs. The prediction of the 3D structure of the B cell epitopes present in the tandem repeat shows a high structural divergence among the closest gp36 orthologs in *E. mineirensis* (UFMG-EV), *E. canis* and *E. chaffeensis*. These structural differences may explain the results obtained by Doyle *et al.*[14] in which neither gp36 nor gp47 reacted with heterologous antisera.

The C + G content of the *gp36* gene of *E. mineirensis* (UFMG-EV) is higher than the rest of the orthologs previously reported (data not shown). The C + G content in specific genes have been used in systematics as support for the classification of organisms [7], and it is known that recombination significantly increases the silent C + G content of a genome in a selectively neutral manner [37].

Although it is well known that *Babesia bovis*, *B. bigemina* and *Anaplasma marginale* are the most common etiological agents transmitted by *R. (B.) microplus* ticks [38], the detection of any species of *Ehrlichia* in *R. (B.) microplus* ticks has been infrequently reported. The first two reports were in China in the Guangxi Autonomous Region in 1999 [39] and Tibet in 2002 [8]; the second in Thailand in 2003 [36] and the latest one in Xiamen, China in 2011 [40]. Except the isolate from Guangxi, *E. canis*[39], the rest share, based on *16S rRNA*, a 99.9% of homology [36,40] and differ from the ehrlichial species previously reported and classified as *Ehrlichia* spp strain Tibet [8]. In the present study, determined by pairwise alignment, the *E. mineirensis* (UFMG-EV) isolated from *R. (B.) microplus* shares 97% of similarity with the *16S rRNA* sequences of the referred species (data not shown). This is the second report of a new *Ehrlichia* spp isolated from *R. (B.) microplus*, but the first to be reported in the American continent. The identification of *E. mineirensis* (UFMG-EV) in *R. (B.) microplus* ticks suggests a potential of infection and transmission of this agent to cattle in the area where infected ticks are present.

## Conclusions

Based on the molecular and phylogenetic analysis of the genes *16S rRNA*, *groEL, dsb* and *gltA* we concluded that the new microorganism isolated from the hemolymph of *R. (B.) microplus* is a new species of *Ehrichia* with new predicted antigenic properties in the gp36 glycoprotein ortholog. Complementary analysis of C + G content in the gp36 orthologs, distant of groESL spacer and hypervariable region of *16S rRNA* supports the fact that *E. mineirensis* (UFMG-EV) is a separate phylogenetic entity.

Further studies should address the question whether *R. (B.) microplus* is a competent vector for this and other *Ehrlichia* species and whether this new organism is an emerging pathogen for cattle or an endosymbiont of *R. (B.) microplus*.

## Competing interests

The authors declare that they have no competing interest.

## Authors' contributions

AC performed the isolation of the genes, the interpretation of the molecular, *in silico* immunological data and drafted the manuscript. EZ performed the *in vitro* cultivation and maintenance of the microorganism at LMU. MFBR isolated the organism from ticks and established it *in vitro*. JAGS performed the *in vitro* cultivation and maintenance of the microorganism at UFMG. JF contributed to design the molecular and phylogenetic analyses. LG contributed to the overall design and supervision of the study. JJV performed the 3D structure prediction and contributed with the epitope analysis. LP developed the conception and design of the study and contributed in drafting the manuscript. All authors critically revised the manuscript and have given final approval of the version to be published.
